# Thermography as a Breast Cancer Screening Technique: A Review Article

**DOI:** 10.7759/cureus.31251

**Published:** 2022-11-08

**Authors:** Manasi B Rakhunde, Shashank Gotarkar, Sonali G Choudhari

**Affiliations:** 1 School of Epidemiology and Public Health, Datta Meghe Institute of Medical Sciences, Wardha, IND; 2 Community Medicine, Jawaharlal Nehru Medical College, Datta Meghe Institute of Medical Sciences, Wardha, IND; 3 School of Epidemiology and Public Health, Community Medicine, Jawaharlal Nehru Medical College, Datta Meghe Institute of Medical Sciences, Wardha, IND

**Keywords:** thermography, mammography, early detection, breast cancer, breast cancer screening

## Abstract

Globally, breast cancer is the most frequently occurring cancer in women and is the reason for more disability-adjusted life years lost than any other type of cancer. Hence, early screening plays a vital role in reducing breast cancer mortality. Although mammography is the standard procedure used for screening and diagnosis of breast cancer, it still has some limitations. Other methods used for screening include ultrasound and clinical breast examination. Despite its limitations, mammography is the gold standard for screening breast malignancy. Another emerging method for screening is thermography. With recent technological advances, breast cancer screening through thermography has demonstrated several advantages over existing modalities. For this review, a literature search was performed using databases such as PubMed, Google Scholar, and ScienceDirect. The keywords searched included breast cancer, early detection, breast cancer screening, mammography, and thermography. This review discusses the benefits of thermography showing that it can be a significant modality for breast cancer screening. The recent developments in thermal sensors, imaging protocols, and computer-aided software diagnostics hold great promise for making this technique a mainstream screening method for cancer. Moreover, the use of artificial intelligence and thermal imaging to detect early-stage breast cancer can provide impressive results. Therefore, thermography will be a promising technology for the early detection of breast cancer.

## Introduction and background

Cancer is the leading cause of death worldwide, resulting in 10 million deaths in 2020 [1]. The most common cancer in 2020 was breast cancer, with 2.26 million cases, followed by lung cancer, with 2.21 million cases, leading to 685,000 deaths globally [2]. It is estimated that one out of nine women will develop breast malignancy in their lifetime, as reported by the National Cancer Institute of Canada [3]. By the end of 2020, 7.8 million women diagnosed with breast malignancy were alive, making it the world’s most extensive cancer [1]. Moreover, one in 12 women is at risk of developing a breast anomaly during their lifetime, as reported by the World Health Organization (WHO) [4]. Thus, early screening of breast cancer has a significant impact on reducing mortality. In every country, breast cancer occurs in females at any age after puberty, but the rates increase in later life. The most affected age group in women is above 50, which is rare in women below 25 [5]. The survival rate in developing countries is around 50-60% due to late detection [6]. Globally, breast cancer is the reason for more disability-adjusted life years (DALY) lost to women than any other cancer type [4].

Overall, 70% of breast cancer cases are detected when the tumor size is over 30 mm [7,8]. A thorough medical examination is essential because breast cancer can present in various ways. Hence, early detection and regular check-ups are crucial in reducing mortality due to the disease. Women with unrelenting abnormalities (generally lasting from one month or more) should undergo standard tests. The two most broadly used breast screening tools are clinical breast examination and mammography [9,10]. However, currently, an alternate technique that can be used for screening is thermography. Thermography captures heat map images of the target surface through an infrared camera, which helps to detect malignancy [11]. With recent technological advancements, thermography, with the help of machine learning, can be used for screening procedures. Thermography proves to be an effective breast cancer screening technique.

This review aims to examine the new emerging modality for breast cancer screening, i.e., thermography, over the pre-existing modality. As mammography is the standard method used for screening, the new technique, thermography, shows effectiveness in breast cancer screening with fewer limitations. This review discusses the benefits of thermography over the pre-existing modalities, along with its significance in the future for early detection of breast tissue malignancy which can help reduce mortality in women due to breast cancer.

## Review

Methodology

The search strategy included searching databases such as Medical Literature Analysis and Retrieval System Online (Medline) via PubMed, Springer, ScienceDirect, and gray literature (including articles published in conferences and Google Scholar) for relevant publications. Keyword searches based on Medical Subject Headings (MeSH) terms included breast cancer, breast cancer screening, early detection, mammography, and thermography. The above terms used logical connectives AND. Among the articles searched, non-English-language articles and articles that did not discuss breast cancer screening, mammography, and thermography were excluded. Articles whose full texts were not available were also excluded. Finally, 41 articles that discussed breast cancer screening, mammography, and thermography were included in the review.

Breast cancer screening techniques

Mammography

Mammography is the current standard and the most used screening procedure for breast cancer. This method uses a low-dose X-ray system to screen the region of the breast and show the difference in density in the image. The proportion of different tissue types within a woman’s breast is represented by breast density [12]. Women are encouraged to undergo routine screening by mammography to enhance early detection.

Limitations of mammography: At this time, mammograms are the best screening tests for breast cancer. However, mammograms also have limitations. For example, when it comes to showing if a woman has developed breast cancer, it is not 100% accurate. About one in every five breast cancer is not found on screening mammograms [13]. Sometimes, even if breast malignancy is present, the mammogram looks normal, showing a false-negative result in one in eight breast cancer cases. Hence, women may have a false sense of security and think they do not have breast cancer when they do. Even if there is no malignancy in the breast tissue, a false-positive mammogram looks abnormal [14]. False-positive results can be due to the presence of dense breast tissue, a history of breast biopsies, or being under medication for estrogen. The odds are the highest of a false-positive finding for the first mammogram [15].

Challenges of mammography: The challenges faced during mammography and their reasons are presented in Table 1.

**Table 1:** Challenges of mammography.

Challenges of mammography	Reason
Affordability	Regular mammography costs may not be affordable to everyone
Age	Mammography may be less effective in younger patients. Additionally, Berrington de González et al. found that “mammography is less effective before age 50” in their mammography screening trial. This might be because younger women have the propensity to have breast tissue that is substantially more dense [16,17]
Breast density	Mammography is unsuitable for women with implants, breast fibro cysts, or dense breasts. For example, it is complex in mammography to differentiate between the two types of tissue because dense breast and cancerous tissue appear white [18]
Rupture risk	With mammography, there is a risk of breaking the envelope of a cancerous tumor because this procedure involves compressing the breast tissue [19]
Radiation exposure	Mammography slightly increases the risk of radiation-induced breast malignancy. Ionizing radiation can significantly impact undifferentiated cells because they are more susceptible to it [20]

Clinical breast examination

A breast examination performed physically by a medical expert is called a clinical breast examination. A clinical breast test is used with a mammogram to screen women with breast cancer. Park et al. [21] reported that the significance of clinical examination lies in its capability to detect cancer that is not detected on mammography. The combination of clinical and self-examination of the breast tends to improve the detection of breast cancer sensitivity (82%).

Thermography

Thermography uses an infrared camera to detect heat emissions from the targeted body region. Digital infrared thermal imaging is the thermography used to diagnose breast cancer. This method shows high accuracy and is a cost-effective form of diagnosis. The concept behind this test is that as cancer cells multiply, they need more oxygen-rich blood for growth. As there is an increase in blood flow to the tumor, the temperature around the tumor also increases. Malignant cells discharge nitric oxide into the bloodstream and cause impairment in the microcirculation [22]. This released nitric oxide, along with the active growth of the cancerous cells, increases blood circulation and temperature in that particular region. Therefore, evaluating these differences in temperature leads to the detection of the malignant region in the breast [23].

In an experiment, Folkman [24] observed the dependence of the growth of a tumor on angiogenesis by transplanting tumor cells into mice. A large amount of blood flows into the vessels connected to cancer, making them warmer than normal blood vessels and making the blood vessel expand and lengthen. Gautherie [25] claimed that the cell discharged heat due to increased metabolic activity. Consequently, this area appears more bright and hot in the thermal image than the surrounding area. Therefore, it leads to accurate temperature values from skin infrared radiation measurements. The breast thermography theory assumes that the breast tissue is free from abnormal processes that emit predictable heat patterns on the skin surface. When physiological processes disrupt the regular pattern, such as vascular disorders or inflammation, it can be collected using sensitive equipment. The recorded amount of energy is converted into a signal of energy that, along with the help of other parameters, is used to analyze the actual body tissue temperature. Thereby, it provides a diagnostic aperture into the functional physiological status of the given body area, as presented for the breast of the patient. The heat discharge from the body provides an accurate diagnosis of early signs of breast cancer [26]. With this information, it is possible to generate a heat graph of the temperature distribution over the region’s surface to be imaged. Initially, such temperature measurement changes were rudimentary, even with a probe directly on the skin. Today, the sensitivity of modern infrared cameras is so high that it is possible to detect temperature differences of up to 0.025°C [27].

History of thermography: Thermography was originally made available as a screening technique in 1956 and was initially well-received. Research by Lawson [28] in 1956 showed an image of a large breast mass exhibiting variation in temperature with the tissues surrounding the breast. Although the method he used is now outdated, this hypothesis has sparked more than two decades of research into the strategies for the improvement of breast temperature measurement as well as a functional screening method. However, in 1977, the thermography technique fell behind other screening tools, i.e., mammography and ultrasound, and the medical community lost interest in this diagnostic method [29]. Thermography was approved in 1982 by the Food and Drug Administration (FDA) to aid in evaluating breast tumors. Despite initial promise, breast thermography fell far behind in the research race for validated mammography procedures [30]. Previously, thermography was limited to techniques such as infrared thermography, plate thermograms, and liquid crystal imaging. Even though these have resulted in an information base that present researchers can build, reliable results for high sensitivity have not been obtained. In these first sessions, the use of breast surface thermography to detect breast tissue abnormalities showed dramatic improvement. Today, technological advancement has increased the sensitivity of the thermography method [31].

Sensitivity of thermography: Early clinical studies conducted from the 1960s to the 1970s showed that the ability to detect breast cancer by thermography was 84-95% for true-positive rates and 6-13% for false-positive rates [22]. In an article, a multi-site observational study in 2020 on the clinical efficacy of thermalytix comprised 470 asymptomatic and symptomatic women who were present for breast health check-ups [32]. All participants underwent the artificial intelligence-based thermograph and standard screening tests with a sensitivity of about 91.02%. It showed a specificity of approximately 82.39% in the detection of malignancy of breast tissues. The performance of thermalytix in breast cancer screening is presented in Table 2.

**Table 2:** Performance of Thermalytix in breast cancer screening [33]

	True positives	False positives	True negatives	False negatives
Overall	71	69	323	7
Symptomatic	62	52	117	7
Asymptomatic	9	17	206	0

**Figure 1:**
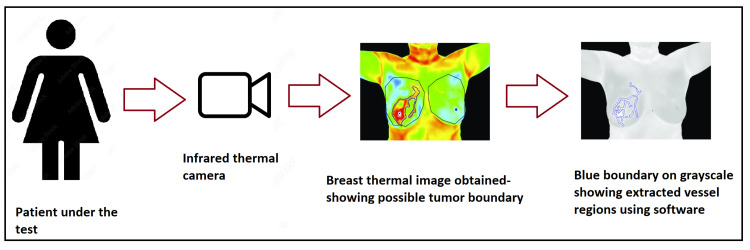
Procedure of breast thermography for screening of breast cancer. This article is available under the Creative Commons CC-BY-NC license and permits non-commercial use, distribution, and reproduction in any medium provided the original work is properly cited [32].

Another test performed in 2020 for personalized risk prediction also used artificial intelligence with thermal screening to generate breast health risk scores [34]. The score was derived based on abnormalities seen in asymmetric heat patterns and others based on vascular activity. The test was performed on 769 women. Out of the 769 women, 185 were already diagnosed with breast malignancy by mammography and ultrasound. This study showed that this method uses breast thermal imaging patterns for assessing malignancy rather than an age-based risk score. Figure 1 shows the procedure of breast thermography for the screening of malignancy.

Advantages of thermography: Thermography is a simple procedure, as simple as taking a picture. Because there is no emission from the camera and nothing is injected into the body, there are no chances of radiation; hence, there is a low chance of damage risk to fragile cellular structures [33]. It is a painless procedure because there is no compression or contact. It is cost-effective (compared with other techniques for diagnostic imaging). It is effective for men, women, and children. Thermography allows the detection of malignancy early and thereby well-timed research and intervention before signs become evident. It is an appropriate device for early health screening for women as it can be effectively used by women of all ages, from pre-adolescence to postmenopausal women. Women of all shapes and sizes can be screened using thermography. Females with dense breast tissue (females in their 20s and 30s), women with fibrocystic cystic breast tissue, and pregnant and breastfeeding females can also be screened. Additionally, screening of menopausal and postmenopausal women is possible [35]. Moreover, women receiving hormone replacement therapy can be screened. Regarding early detection, thermography outperforms other modalities. Thermography can detect changes such as vasodilatation, angiogenesis, and severe flexion of blood vessels that can be seen in early cancer stages [36]. Using machine learning and artificial intelligence-based thermography for screening reduces human mistakes. Still, these methods do not make these mistakes as they make decisions by previously gathered information using algorithms. Another advantage of this method is that it performs procedures faster than humans. The thermography method allows large-area assessments and real-time detection-making.

Disadvantages of thermography: The problem with the thermography test is that it is difficult to distinguish the cause of the increase in heat. The warm part of the breast may be a sign of breast cancer, but it may also indicate a non-cancerous condition such as mastitis [37]. Because when there is inflammation in the breast due to bacterial or viral infection, the temperature of the tissues increases, affecting the thermography results.

Recent advances: Recently, in India, Niramai’s Thermalytix solution has been developed. It is based on identifying highly active cancer cells that create a higher temperature region by enlargement or dilation of blood vessels and forming new vascular lesions. NIRAMAI stands for Non-Invasive Risk Assessment by Machine Intelligence [38]. This automated breast health check and diagnosis tool combines thermal imaging with artificial intelligence. The infrared camera is handy and relatively low cost compared to two-dimensional mammography equipment. With the help of this imaging procedure, the temperature variation shows the heat pattern in the breast tissue, which the thermalytix artificial intelligence algorithm analyzes to detect temperature variations [39].

**Figure 2:**
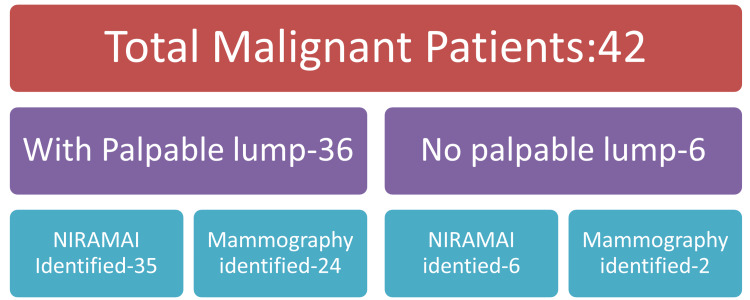
Effectiveness of mammography and NIRAMAI as a screening test. This article is available under the Creative Commons CC-BY-NC license and permits non-commercial use, distribution, and reproduction in any medium provided the original work is properly cited [40].

A comparative study was performed on 147 patients who visited a cancer hospital in Bengaluru with breast complaints or preventive measures. On analysis, thermalytix showed 98% sensitivity and specificity over 68%. Thermalytix detected all malignant cases, including patients with no palpable breast lumps [40]. Out of 147 patients, 42 were found to have malignancy by performing mammography and sono-mammography. Among these patients, 14% were under 40 years of age. Moreover, 93% had breast symptoms. The high sensitivity of this modality in any age group of women shows its effectiveness in screening breast cancer. Thermalytix, with the help of the automatic scoring and image interpretation of potential vascularity and malignancies, can help clinicians make better decisions and improve care quality in a radiation-free and affordable manner. Consequently, thermography has advanced significantly to match mammography’s level of accuracy [41]. Thus, it is believed that the thermalytix technique is on edge to be a potential approach for screening breast cancer. Figure 2 shows the effectiveness of the thermalytix NIRAMAI compared with mammography.

Table 3 shows a comparison between mammography and thermography as screening tools for breast cancer.

**Table 3:** Comparison between mammography and thermography. [14,16,23,33].

Parameters	Mammography	Thermography
Checks for	Anatomical abnormality	Physiological abnormality
Involves detection of	Lymph nodes in the neck, breasts, and under the arms	Unable to detect lymph nodes in the neck and under the arms
Affordability	Costly, not affordable to everyone	Cost-effective
Age	Less effective in women of younger age	No age barrier
Having a history of mastectomies	Not effective in women who have undergone mastectomies	Ideal for women who have undergone mastectomies
Women with implants	Not as effective for women with implants	Effective
Breast density	Can show the false-positive result in women having dense breast tissue	Breast density does not affect the results
Further investigation	Results in unnecessary biopsies for women with dense and fibrocystic tissues	Fewer unnecessary biopsies
Pain and fear	Compression of the breast involved can be painful and cause fear	No compression is involved
Rupture risk	Involves compression of the breast, hence, the risk of rupturing the tumor envelope	No risk of rupture
Radiation exposure	Uses ionizing radiation.	Does not involve radiation.
Food and Drug Administration (FDA) approval	FDA approved for breast cancer diagnosis	FDA approved as an adjunct to mammography

Just as a fingerprint is unique, each patient has a specific infrared map of the breast tissue. Moreover, any refinement of this infrared map on the images is taken for months to years. It can consist of an early sign of abnormality. In patients without cancer, these examination results are used to monitor the possibility of cancer risk in the future. By monitoring the patient’s breast health with the help of infrared imaging, self-breast examinations, clinical examinations, etc., a woman can have a much better possibility of detecting the malignancy in tissues on time, preventing the invasive growth of the tumor and leading to the severity of the disease.

## Conclusions

Despite not providing morphological information regarding the breast, thermography provides valuable data on the thermal and circulatory characteristics of the tissue. Studies suggest the potential usefulness of thermography in the early detection of breast abnormalities that may develop into cancer because the physiological changes in tissue occur prior to pathological changes. Thus, thermography is very useful in avoiding unnecessary biopsies in the case of benign breast cancer and identifying early cases of breast cancer. Currently, no screening method can predict the presence of a malignant tumor with 100% accuracy. The only reliable diagnostic method is a biopsy. However, with the encouraging accuracy and thermography’s fundamental advantages of low cost, portability, and painless technique, it can be a viable alternative to the currently used mammography. Still, in clinical practice, the fundamental role of thermography needs to be evaluated through a large multicenter trial that would estimate the digital thermography accuracy in breast cancer screening. It is suggested to perform thermography as a complementary tool in the clinical examination of the breast. Thermography can have a significant impact on developing countries, where there is less availability of healthcare workers. Due to the low cost involved in thermography, communities with limited resources will benefit from providing the modality for early breast malignancy detection. Early cancer identification will reduce the burden on undeveloped communities with inadequate health infrastructure.
